# Mouse embryo phenotyping using X-ray microCT

**DOI:** 10.3389/fcell.2022.949184

**Published:** 2022-09-16

**Authors:** Stephan Handschuh, Martin Glösmann

**Affiliations:** VetCore Facility for Research / Imaging Unit, University of Veterinary Medicine Vienna, Vienna, Austria

**Keywords:** μCT, X ray microtomography, growth and development, laboratory animal models, fetus

## Abstract

Microscopic X-ray computed tomography (microCT) is a structural *ex vivo* imaging technique providing genuine isotropic 3D images from biological samples at micron resolution. MicroCT imaging is non-destructive and combines well with other modalities such as light and electron microscopy in correlative imaging workflows. Protocols for staining embryos with X-ray dense contrast agents enable the acquisition of high-contrast and high-resolution datasets of whole embryos and specific organ systems. High sample throughput is achieved with dedicated setups. Consequently, microCT has gained enormous importance for both qualitative and quantitative phenotyping of mouse development. We here summarize state-of-the-art protocols of sample preparation and imaging procedures, showcase contemporary applications, and discuss possible pitfalls and sources for artefacts. In addition, we give an outlook on phenotyping workflows using microscopic dual energy CT (microDECT) and tissue-specific contrast agents.

## 1 Background

The mouse is the most common used model for studying mammalian gene function. However, about 30% of all knockout mouse lines are embryonic or perinatal lethal. In these lines, linking function of genes to phenotypes cannot be accomplished by analysis of adult individuals but requires investigation of development (Wong et al., 2014). As part of an ambitious effort of characterizing 20.000 knockout mouse lines, the International Mouse Phenotyping Consortium (IMPC) came up with recommendations for a high-throughput pipeline using 3D imaging techniques to search for previously unknown embryonic phenotypes (Adams et al., 2013; Wong et al., 2014). Subsequent screening efforts of the IMPC involved several 3D imaging techniques, including optical projection tomography (OPT) for early developmental stages (E9.5), microscopic X-ray computed tomography (microCT) for later developmental stages (E15.5, E18.5), high-resolution episcopic microscopy (HREM) to reveal histological detail, and high-resolution magnetic resonance imaging (micro-MRI) for postnatal brain imaging (Dickinson et al., 2016). Among this portfolio of imaging modalities, microCT contributed remarkably to the identification of unknown phenotypes (Wong et al., 2014; Dickinson et al., 2016). In addition, microCT has been used as key imaging modality in numerous standalone projects that required the structural analysis of parts of the embryo such as the cardiovascular system (Degenhardt et al., 2010; Kim et al., 2013). This review provides an overview of the complete workflow of microCT analysis from fixation of embryos to the acquisition of tomographic datasets. We further exemplify contemporary applications of microCT imaging for mouse embryo phenotyping, including qualitative and quantitative analysis of mutant phenotypes, as well as fully automated computer-based detection of novel phenotypes.

## 2 Microscopic X-ray computed tomography

MicroCT delivers genuine and isotropic 3D information from dense and non-transparent biological samples at micron resolution. MicroCT data is calibrated for both geometry and intensity, making microCT a powerful tool especially for quantitative studies. Over the last two decades, microCT has gradually evolved into a routine technique also in many biomedical research disciplines. More detailed accounts of the principles of X-ray imaging, technical design of microCT systems, and image formation are given elsewhere (Ritman, 2004, 2011; Mizutani and Suzuki, 2012; de Chiffre et al., 2014; Maire and Withers, 2014; Rawson et al., 2020; Stock, 2020; Kalender, 2021). In short, the specimen (e.g., embryo in agarose inside a polypropylene tube) is mounted on a sample holder and put on a 360° rotation stage located between the X-ray source and X-ray detector inside the microCT scanner (Figure 3A). During the scan, the specimen rotates in tiny increments about its vertical axis, and a sequence of X-ray projection images is acquired as raw tomography data. Like in conventional radiography, contrast in projection images is based on differences in X-ray attenuation between different parts of the sample. From the recorded raw data, a series of virtual sections is reconstructed using dedicated computer algorithms. The reconstructed image volume (image stack, series of tomograms) is calibrated in terms of geometry and image intensity values, and the spatial resolution is typically isotropic (same image resolution in X-Y-Z).

Commercial laboratory microCT systems use polychromatic X-ray sources and filtered or unfiltered spectra at peak voltages typically varying between 40 kVp (Metscher, 2009b) and 100 kVp (Wong et al., 2014) for imaging of mouse embryos. With benchtop scanners, scan acquisition time is usually in the range of several hours (e.g., 5 h, Wong et al., 2014)). Benchtop scanners are quite affordable and in many systems, several samples can be queued for scanning. This means that even with commercial benchtop scanners a high sample-throughput can be achieved as demonstrated by the IMPC screening for novel developmental phenotypes (Dickinson et al., 2016). With synchrotron X-ray imaging beamlines, scan acquisition times can be shortened to minutes or even seconds (Maire and Withers, 2014) thus further dramatically increasing sample throughput. However, access to synchrotron beamlines is quite limited compared to lab-based scanners. In future, laser-driven X-ray sources may allow to image embryos at high resolution in minutes instead of hours even in the laboratory (Cole et al., 2018).

**FIGURE 1:**
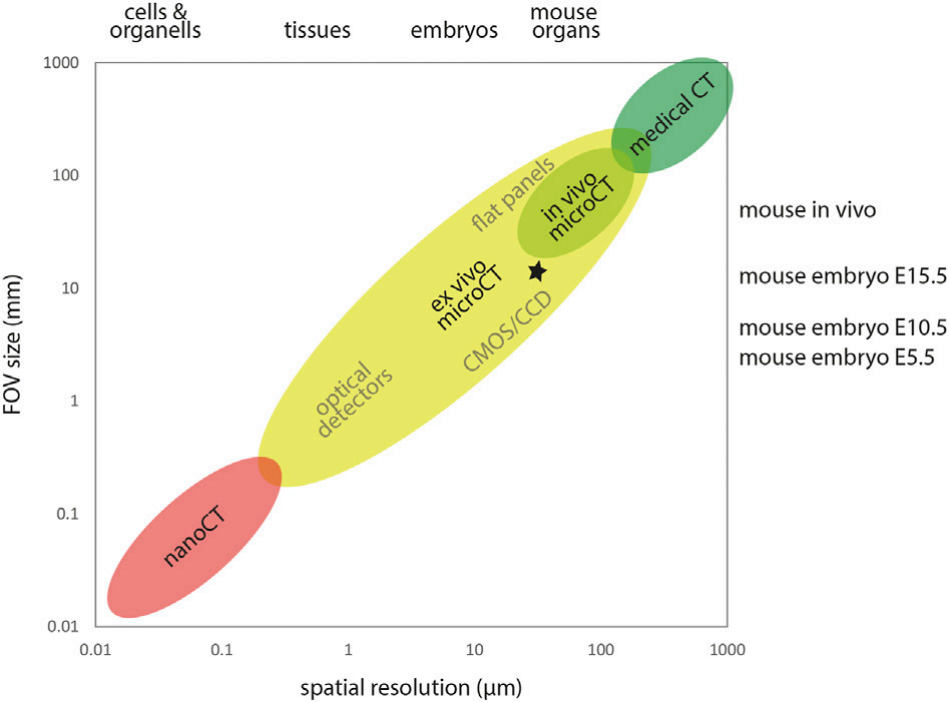
X-ray computed tomography across scales. X-ray computed tomography covers a wide range of samples sizes and spatial resolution. Detector size and FOV typically constrain the achievable spatial resolution. MicroCT encompasses FOVs from roughly 0.3–300 mm, delivering spatial resolution in the micron range. In the high throughput IMPC screening (Dickinson et al., 2016), E15.5 embryos were imaged using a lateral FOV of ∼13 mm and an isotropic voxel size of ∼13 µm, yielding a spatial resolution of ∼30 µm in reconstructed image volumes (asterisk).

Spatial resolution in microCT images is strongly linked to sample size and field of view. X-ray computed tomography can be used across multiple scales ranging from microscopic to the size of human patients. As a rule of thumb, image resolution is 1/1000 of the imaged field of view (Figure 1). For example, imaging of E15.5 embryos within the IMPC screen was performed with a lateral FOV of approximately 13 mm for reconstructing virtual slices of 1000 × 1000 pixel at an isotropic voxel size of approximately 13 µm (Wong et al., 2014; Dickinson et al., 2016), yielding a spatial resolution of approximately 30 µm. Gabner et al. (2020) imaged E16.5 embryos with a lateral FOV of approximately 18 mm for reconstructing virtual slices of 2000 × 2000 pixel at an isotropic voxel size of approximately 9 µm, yielding a spatial resolution of approximately 20 µm. Earlier embryos can be imaged at much smaller voxel sizes and thus higher resolution. Ermakova et al. (2018) imaged E7.5 embryos with an isotropic voxel size of 1.4 µm and E8.5, E9.5, and E10.5 embryos with an isotropic voxel size of 3.9 µm. The enormous flexibility of microCT with respect to accessible sample size allows investigation of developmental stages of small (e.g., mouse) and large animal models (e.g., horse, Okada et al., 2020; Scarlet et al., 2021) equally well using this modality.

## 3 Preparation of mouse embryos for microCT imaging

### 3.1 Fixation

Adequate sample preparation is key to the acquisition of meaningful microCT image data. Scan times for investigating embryonic phenotypes are up to several hours during which the specimen must not move. In addition, the micron-level resolution of microCT allows resolving structural deformation by tissue disintegration during scanning. Therefore, similar to classical histology, chemical fixation is mandatory to preserve embryo fine structure in microCT studies. Particularly in correlative workflows and large-scale morphological screening projects, it is relevant to match fixation with all post-fixation requirements of sample preparation to avoid effects of incompatibility of reagents and ensure conditions of identical processing as a prerequisite for qualitative and quantitative comparative analysis. Widely in microCT analyses, neutral buffered 4% formaldehyde (NBF) is used for mouse embryo general structural preservation, as it combines fast tissue penetration with effective cross-linking of proteins, prevents excessive tissue shrinkage and deformation, and is compatible with standard histopathological preparation protocols that may follow microCT analysis. Fixation of embryos in the standardized IMPC phenotyping pipeline is in 4% formaldehyde made from depolymerized paraformaldehyde (PFA) in phosphate buffered saline (PBS), to provide for a reproducible concentration of formaldehyde in buffer (Wong et al., 2014; Dickinson et al., 2016). Early morphometric studies used fixation in Bouin’s solution, a compound composed of picric acid, acetic acid, and formaldehyde, to enhance embryo rigidity for scanning specimens in air (Boughner et al., 2008). However, Bouin’s causes inadequate tissue shrinkage and shape deformation (Schmidt et al., 2010), making it an undue fixative particularly for quantitative analyses. The use of other than formaldehyde-based fixatives is possible and frequently required in specific contrast staining protocols to ensure compatibility of reagents, e.g., ethanol in combination with ruthenium red staining of embryonic cartilage (Gabner et al., 2020). Taken together, different fixatives are in use for microCT analysis of mouse embryos and differentially affect sample properties relevant to morphometric and biochemical analysis. This needs to be considered in the interpretation of experimental findings (see also 5.3).

### 3.2 Embryo staining with contrast agents

**FIGURE 2:**
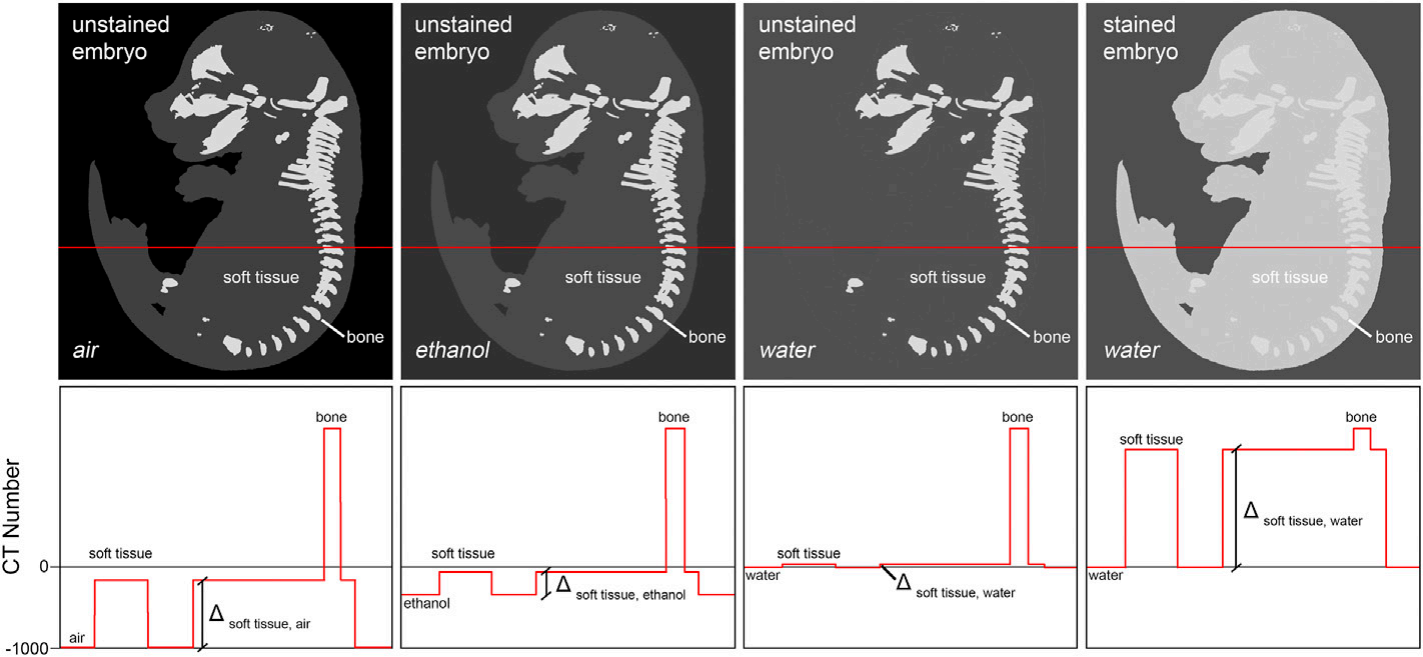
Achieving soft tissue contrast in microCT imaging. Fixed but otherwise untreated, non-mineralized embryo tissue shows only little difference in x-ray attenuation to aqueous mounting media. Thus, its imaging in aqueous media is not applicable. One possibility to increase soft tissue contrast is infiltration of the tissue with a contrast agent containing a high atomic number (Z) compound, thus increasing X-ray attenuation of tissue relatively to the mounting medium (‘staining’). Alternatively, it is possible to decrease X-ray attenuation in the mounting medium by replacing water with ethanol or paraffin, or by drying the embryo followed by imaging of the sample in air. Of all approaches, staining typically yields highest soft tissue contrast.

In the mouse embryo, only the mineralized skeleton shows significant inherent X-ray contrast (Oest et al., 2008). Other embryonic tissue is nearly transparent to the X-ray beam when imaged in aqueous environment. Thus, sample preparation for whole embryo imaging needs to deploy means to increase soft tissue contrast. Two different methods are commonly used to provide for adequate signal to background ratios. The first technique increases contrast by enhancing intrinsic X-ray attenuation of the embryo through infiltration with an X-ray dense contrast agent (‘staining’). Typically, contrast agents contain compounds with atomic numbers (Z) higher than 35 (bromine, e.g., in eosin Y (Busse et al., 2018)) in order to effectively increase tissue contrast. A second technique reduces image background by replacing water in or around the sample with a medium of lower X-ray attenuation such as ethanol, paraffin, or air, to increase overall specimen contrast (Figure 2).

#### 3.2.1 Non-specific tissue contrast

**FIGURE 3:**
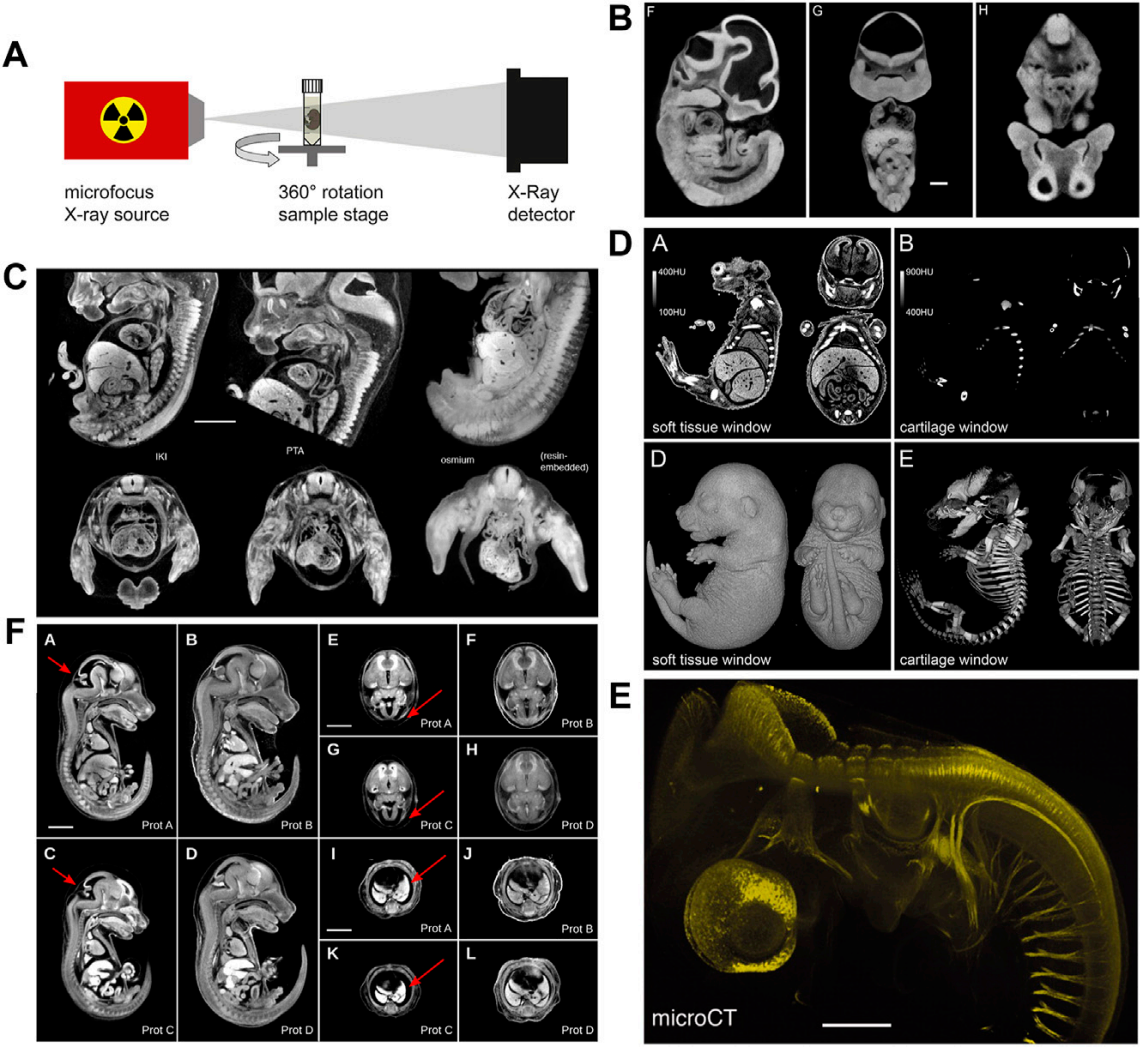
MicroCT image acquisition and examples of non-specific and tissue-specific staining. **(A)** Schematic representation of a micro-CT system comprised of an X-ray source and an X-ray detector coupled to a camera. Samples are mounted to a rotating stage between the source and the detector (Figure 1F from Gabner et al., 2020, Development 147 (11)). **(B)** MicroCT image contrast derives from differences in X-ray attenuation between different tissues, achieved by staining with X-ray dense contrast agents. Osmium tetroxide delivers non-specific contrast to embryonic soft tissue enabling the visualization of structural features (Figures 1F–H from Johnson et al., 2006, PLoS Genetics 2 (4). **(C)** Mouse embryos stained with iodine (I_2_KI), phosphotungstic acid (PTA), and osmium tetroxide. The different non-specific contrast agents show subtle differences in absorbance levels between tissues (Figure 9 from Metscher 2009a). **(D)** Selective cartilage staining with ruthenium red enables the simultaneous visualization of cartilage and bone in the developing mouse skeleton (Figures 3A,B,D,E from Gabner et al., 2020, Development 147 (11)). **(E)** Selective antibody labelling of acetylated α-tubulin in a stage 23 chicken embryo. Antibody binding sites visualized by microCT after horseradish peroxidase-mediated deposition of X-ray dense atomic silver [parts of Figure 1 from Metscher and Müller, 2011, Developmental Dynamics 240 (10)]. **(F)** Hydrogel-embedding of mouse embryos prior to iodine staining significantly reduces shrinkage artefacts. Prot. A embryo stained with 0.025N I_2_KI without pre-treatment, Prot. B embryo pre-treated with hydrogel and stained with 0.025N I_2_KI, Prot. C embryo stained with 0.1N I_2_KI without pre-treatment, Prot. D embryo pre-treated with hydrogel and stained with 0.1N I_2_KI (Figure 3 from Wong et al., 2013b, PLoS ONE 8 (12). **(A and D)** reprinted by permission from the Company of Biologists. **(E)** reprinted by permission from John Wiley and Sons. **(B,C and F)** reprinted under the terms of the Creative Commons Attribution License.

Historically, osmium tetroxide has been first used to contrast soft tissue for microCT phenotyping of formalin-fixed mouse embryos (Johnson et al., 2006). Osmium tetroxide reacts with lipids and is used as a secondary fixative in electron microscopy (Woods and Stirling, 2008). Johnson et al. (2006) using osmium tetroxide-staining and microCT imaging yielded virtual histology datasets of transgenic mouse embryos (Figure 3B) that allowed phenotyping based on image segmentation, as demonstrated for organs such as brain, liver, and heart. Subsequently, several other high-Z compounds including elemental iodine (I_2_), iodine potassium iodide (I_2_KI), and phosphotungstic acid (PTA) have been rigorously explored by Metscher (2009a, 2009b) for their suitability to contrast vertebrate embryos. Both iodine and PTA provide soft tissue contrast comparable to osmium tetroxide (Figure 3C), and protocols spread rapidly in the community since these compounds are cheaper and less toxic. Particularly, iodine staining is versatile (Metscher, 2009a) and thus became widely used for contrasting microCT samples in research fields even beyond developmental biology (Gignac et al., 2016). It was later shown that in mouse embryos, phosphomolybdic acid (PMA) yields contrast similar to PTA (Descamps et al., 2014). Between 2010 and 2020, iodine and PTA were the most commonly used contrast agents in microCT studies visualising embryo morphology. Overall, in this decade, the prospects offered to the research field of developmental biology by contrast-enhanced microCT imaging caused remarkable excitement.

#### 3.2.2 Tissue specific contrast agents

Only recently, a series of studies established the usability of X-ray suitable staining agents to specifically contrast tissues, such as a hematein lead (II) complex (Müller et al., 2018) and lead (II) acetate (Metscher, 2020) for cell nuclei and eosin Y (Busse et al., 2018) for cytoplasm. Gabner et al. (2020) presented a protocol for staining embryonic cartilage in whole mounts using ruthenium red, thus, for the first time allowing to simultaneously image cartilage and bone in the developing mouse skeleton in 3D (Figure 3D). Specific targeting of tissue components is possible as well. Metscher and Müller (2011) showed that microCT could be utilized for imaging of molecular signals (Figure 3E). Their approach was based on whole-mount immunostaining using a peroxidase-conjugated secondary antibody, followed by enzyme-mediated silver deposition.

#### 3.2.3 Tissue distortions and technical refinements of staining procedures

Tissue volume changes are well documented for standard histological processing and also occur during chemical fixation, dehydration, and drying or paraffin embedding for microCT sample preparation. Several studies demonstrated severe shrinkage of adult mouse and rabbit tissue after employing I_2_KI, I_2_, and PTA staining protocols (Vickerton et al., 2013; Buytaert et al., 2014; Heimel et al., 2019) suggesting that these protocols are of limited use for quantitative studies of soft tissues, including embryos. This problem was overcome by the STABILITY protocol (Wong et al., 2013b), which stabilizes embryonic tissues by embedding embryos in an acrylamide hydrogel matrix prior to staining, thus substantially reducing shrinkage and inter-sample variation in iodine-stained embryos (Figure 3F). The STABILITY protocol was used later in studies of normal mouse development (Hsu et al., 2016) and in the IMPC high-throughput screenings of mouse mutants (Wong et al., 2014; Dickinson et al., 2016). More recently, it was demonstrated that the commercial X-CLARITY^TM^ hydrogel solution similarly reduces shrinkage in early post-implantation stages (Hsu et al., 2019).

### 3.3 MicroCT imaging of unstained embryos

MicroCT imaging of fixed mouse embryos is possible even without contrast agents. Nagase et al. (2008) avoided post-fixation with highly toxic osmium tetroxide and after fixation processed samples with hexamethyldisilazane, which is used in SEM processing as an alternative to critical-point drying, followed by imaging of dry specimens in air. As mentioned above, fixation in Bouin’s solution and imaging the sample in air yields adequate contrast at the embryo surface (Boughner et al., 2008; Parsons et al., 2008). While Bouin’s fixation provides sufficient tissue stiffness to allow scanning of fixed wet embryos in air without movement artefacts, it was found to cause shrinkage artefact inacceptable for many types of morphometric examination (Schmidt et al., 2010). Therefore, for volumetric analyses of organ development, fixation in Bouin’s was abandoned in later studies and instead critical point drying and imaging in air employed for the imaging of unstained mouse (Brosig et al., 2018) and rat embryos (Markel et al., 2021). Paraffin embedding also reduces image background compared to imaging in aqueous media and therefore has been used to image embryos without prior contrast staining (Ermakova et al., 2018). However, similar to fixation in Bouin’s, critical point drying and paraffin embedding are known to cause severe tissue shrinkage (Boyde and Maconnachie, 1979; Tran et al., 2015; Rodgers et al., 2021), thus limiting their use for quantitative studies.

### 3.4 Sample mounting

To prevent specimen movement during image acquisition, a stable sample mounting is essential. Typically, mouse embryos are mounted in plastic containers. However, fragile embryonic tissue cannot be wrapped in gauze as frequently is done with stiffer samples such as bone. Therefore, mouse developmental stages are commonly mounted in agarose. Low melting temperature agarose allows for convenient positioning of the specimen when the agarose is fluid at 30–35°C and provides stable support for the sample after hardening to a gel at room temperature, at which scanning is performed (Metscher, 2011; Wong et al., 2013b; Dickinson et al., 2016; Hsu et al., 2016; Cole et al., 2018; Hsu et al., 2019; Gabner et al., 2020). Alternatively, for scanning in liquid environment, embryos can be placed in polypropylene microfuge tubes (Johnson et al., 2006) or pipette tips (Metscher, 2009b; Fink et al., 2020). Lesciotto et al. (2020) mounted PTA-stained embryos in a 1:1 mix of polyester wax and paraffin to avoid sample movement and desiccation. For imaging of embryos inside the uterus, whole iodine-stained uteri have been mounted in narrow plastic columns (Ermakova et al., 2018). The size of the plastic container used for mounting should match specimen size, because excess medium such as PBS, agarose, or ethanol decreases the signal-to-noise ratio in projection images, particularly in samples with low intrinsic contrast. The adverse effect of excess paraffin coating on soft tissue contrast of unstained horse embryos has recently been demonstrated (Handschuh et al., 2022). Another important aspect in specimen mounting is the proper sealing of the mounting container. Inside the microCT scanner, the temperature is slightly higher than room temperature. Mounting medium could evaporate during scanning and cause specimen movement, specimen desiccation, or contamination of the microCT scanner. Containers therefore need to be closed (microfuge tubes with plastic caps) or sealed with parafilm (pipette tips).

## 4 MicroCT for imaging mouse development

Modern 3D imaging methods including microCT show three major advantages over traditional histology-based approaches for embryo phenotyping. First, they offer faster image acquisition and the rapid availability of complete three-dimensional datasets accelerating the qualitative inspection and analysis of specimens. Second, 3D images provide a basis for different kinds of quantitative analysis such as volumetry, morphometry, geometric morphometrics, and densitometry. Third, 3D digital data can be utilized for automated computer-based recognition of phenotypes that are too subtle to be detected by eye.

### 4.1 Qualitative analysis

Compared to traditional serial sectioning approaches, reconstruction of whole embryo structure using modern 3D imaging instrumentation such as microCT is faster and, more importantly, sample preparation is less time-consuming in terms of working hours. Furthermore, image data can be inspected and analysed by taking advantage of the wealth of commercial and non-commercial 3D visualisation software packages. This facilitates our understanding of the complex 3D organisation of structures *in situ* and makes it easier to detect developmental abnormalities compared to two-dimensional tissue sections.

**FIGURE 4:**
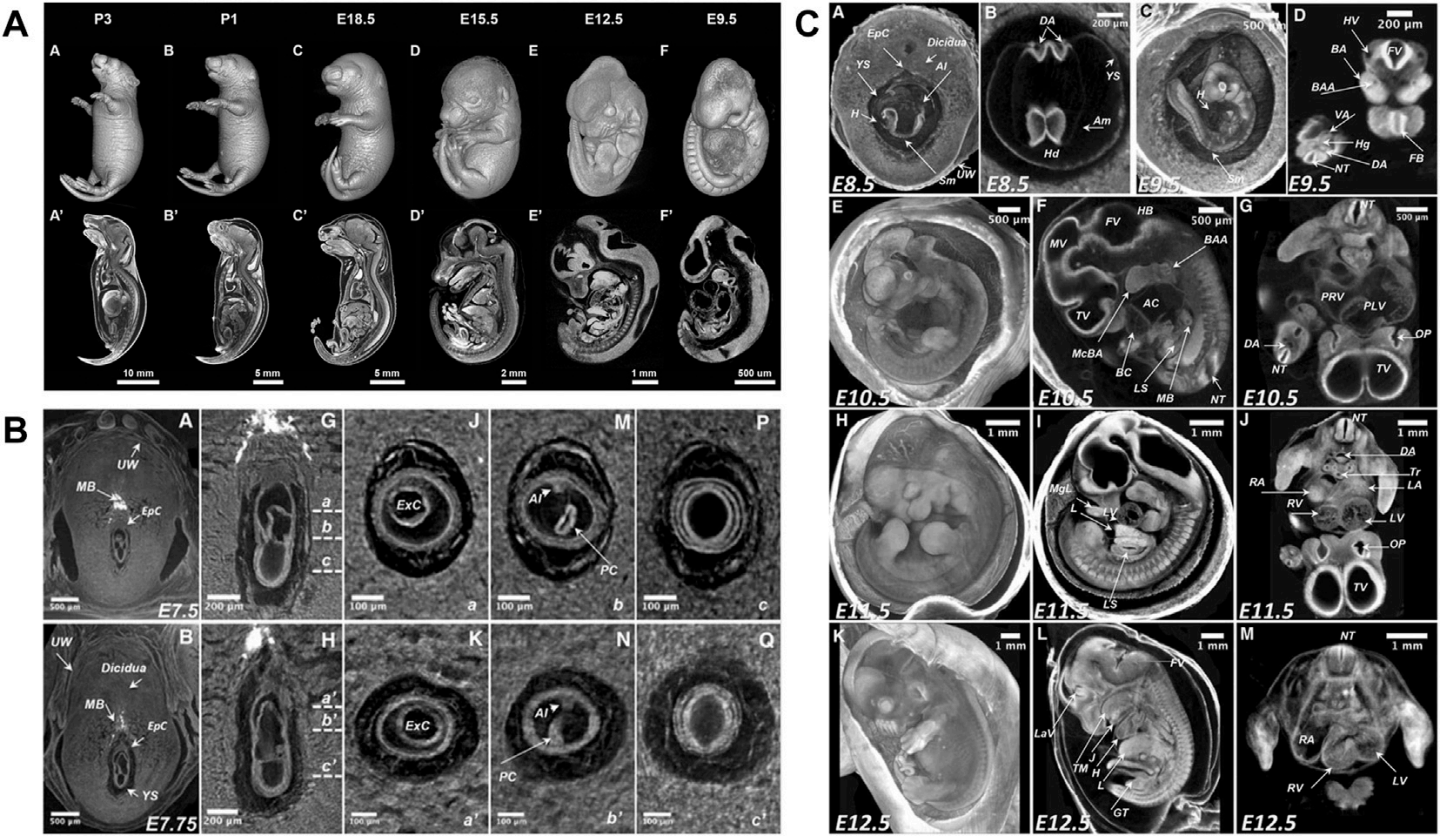
MicroCT slices from volume datasets and volume renderings of the developing mouse at selected stages. **(A)** Documentation of normal mouse development from postimplantation to early postnatal stages after aldehyde fixation and iodine staining [Figure 1 from Hsu et al., 2016, Developmental Biology 419 (2)]. **(B)** Detailed depiction of the gastrulation period around E7.5 (parts of Figure 3 from Ermakova et al., 2018, Mammalian Genome 29). **(C)** Mouse developmental stages from E8.5 to E 12.5 including turning of the embryo between E8.5 and E9.5 (Figure 5 from Ermakova et al., 2018, Mammalian Genome 29). All images reprinted under the terms of the Creative Commons Attribution License.

The detailed knowledge of normal mouse development and its variability is prerequisite for the detection of novel phenotypes. Traditionally, reference atlases of normal development derived from conventional histological examination (Theiler, 1989; Kaufman, 1994) and later became available as online resources (Graham et al., 2015). Likewise, microCT studies aimed at documenting sequences of normal mouse development to serve as a reference for the detection of new phenotypes. Hsu et al. (2016) reported mouse development from E8.5 to early postnatal (P3) (Figure 4A), while Ermakova et al. (2018) described stages from immediate post-implantation (E5.5) to mid gestation (E12.5) including gastrulation phase around E7.5 and turning of the embryo around E9 (Figures 4B,C). Image plates and videos in these papers provide a suitable reference for comparison. As a next step, a microCT-based free online atlas of complete mouse development would serve as a valuable reference for future studies.

Other microCT studies did not focus on the whole embryo, but instead covered only specific aspects of normal development. Degenhardt et al. (2010) reported data on heart development at E11.5 and E13.5, and showed that for newborn pups the iodine-stained blood cells can be used for a 3D angiography. Oest et al. (2008) described the development of mineralized tissue (bone and teeth) from E17 to E19. Aoyagi et al. (2012) presented data for Meckel´s cartilage, the otocyst, and the tongue musculature at E13. Khonsari et al. (2013) presented a 3D model of the developing pituitary gland for E11.5. Matula et al. (2021) reported and made publicly available data for cranial development between E12.5 and E18.5. Gabner et al. (2020) depicted the entire developing skeleton at E16.5. In addition to these studies on embryos and fetuses, De Clercq et al. (2019) investigated and quantified the three-dimensional structure of the mouse placenta.

**FIGURE 5:**
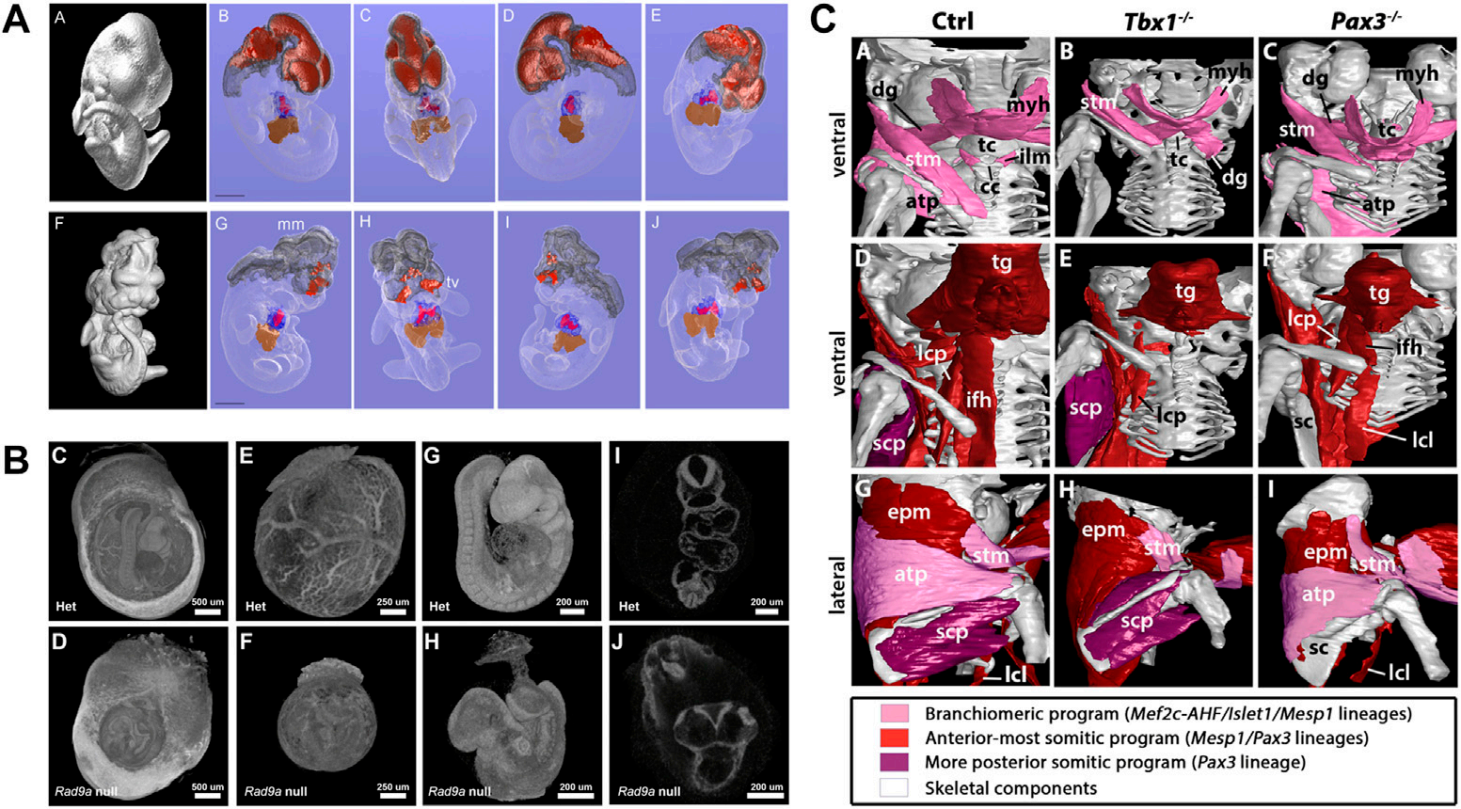
MicroCT for qualitative analysis of mutant mouse embryos. **(A)** MicroCT data used for isosurface and segmentation renderings to facilitate comparison of wildtype (top) and *Pax3:Fkhr* oncogene expressing (bottom) E11.5 mouse embryos [Figures 4A–J from Johnson et al., 2006, PLoS Genetics 2 (4)]. **(B)** Volume renderings of heterozygous and embryonically lethal *Rad9a* null mice at E9.5. Embryos imaged within yolk sacs and digitally segmented out from the original data set to assess early and subtle structural alterations unaffected by preparation artefacts (Figures 4C–J from Hsu et al., 2016, Developmental Biology 419 (2)). **(C)** Segmentation and surface renderings allow depiction of musculoskeletal malformations in *Tbx1* and *Pax3* mutants (Figure 6 from Heude et al., 2018, eLife 7). All images reprinted under the terms of the Creative Commons Attribution License.

Several studies qualitatively analysed developmental malformations based on visual inspection and comparison to normal development (summarized in Table 1). Johnson et al. (2006) were the first to use microCT to compare wildtype mouse embryos to mutants based on segmentation and 3D renderings, showing several developmental defects in mutants expressing *Pax3:Fkhr* in neural crest and myogenic tissue including partial neural tube closure failure and severely hypomorphic telencephalic vesicles (Figure 5A). Nagase et al. (2008) investigated two embryonic models for craniofacial anomalies (holoprosencephaly and fetal alcohol syndrome) at E11 based on virtual slices and 3D renderings. Several studies addressed congenital heart disease models. Degenhardt et al. (2010) investigated cardiovascular defects in *PlexinD1* mutants at E17.5, confirming previously reported defects such as a ventricular septal defect and identifying a previously unknown abnormal cardiac venous connection. Tobita et al. (2010) showcased the usability of microCT to detect pathologies including polydactyly, cleft palate, and situs inversus. Kim et al. (2013) reported congenital heart defects including ventricular septal defects and outflow tract anomalies in fetuses and stillborns obtained from the breeding of N-ethyl-N-nitrosourea (ENU) mutagenized mice. Hsu et al. (2016) showed several defects for embryonically lethal *Rad9a* null embryos at E9.5, including failure of remodelling of the yolk sac vasculature and failure of embryo turning, and still open anterior neural folds, among several other dysmorphic features (Figure 5B). Open anterior neural folds together with severe growth retardation were also reported by Fink et al. (2020) for homozygous *Ing3* knockouts at the time of embryo death (E10.5). MicroCT has also been used to support determination of the exact developmental window of embryonic lethality. Ermakova et al. (2018) investigated a mouse line with knockout for tRNA splicing endonuclease subunit *Tsen54* gene. Using whole-uterus imaging, they found that development of *Tsen54* null animals is arrested at peri-implantation period between E4.5 and E5.5. Heude et al. (2018) showed different types of malformations of the neck musculoskeletal system in *Tbx1* and *Pax3* mutants (Figure 5C).

**TABLE 1:** Overview of microCT studies investigating disorders in mouse organ development.

	qualitative studies	quantitative studies
cranial skeleton		Jones et al. (2010), Rafiq et al. (2012), Hill et al. (2014), Ho et al. (2015), Kawasaki et al. (2017), Marghoub et al. (2018), Kwon et al. (2018)
post-cranial skeleton		Zhang et al. (2009), Moncayo-Donoso et al. (2019)
musculo-skeletal	Heude et al. (2018)	
craniofacial shape and cleft	Nagase et al. (2008), Tobita et al. (2010)	Parsons et al. (2008), Boughner et al. (2008)
cardiovascular system	Degenhardt et al. (2010), Kim et al. (2013), Desgrange et al. (2019)	Merchant et al. (2016)
central nervous system	Johnson et al. (2006), Fink et al. (2020)	Muha et al. (2021)
lung	Desgrange et al. (2019)	
abdominal organs	Desgrange et al. (2019)	Desgrange et al. (2019), Muha et al. (2021)
situs inversus	Tobita et al. (2010)	
polydactyly	Tobita et al. (2010)	

### 4.2 Quantitative analysis

MicroCT has also been extensively used to quantitatively characterize developmental abnormalities (summarized in Table 1). Several early studies examined the shape of embryos using geometric morphometric tools such as Procrustes analysis. Parsons et al. (2008) analysed the cranial dysmorphology of A/WySn embryos, a model for cleft lip and palate, between E10.5 and E11.5. Boughner et al. (2008) investigated the facial shape of *crf4* mutants that exhibit a significant reduction in brain size and basicranial length in adult mice, showing that aspects of the Crf4 phenotype are already evident in embryos between E10 and E12.

**FIGURE 6:**
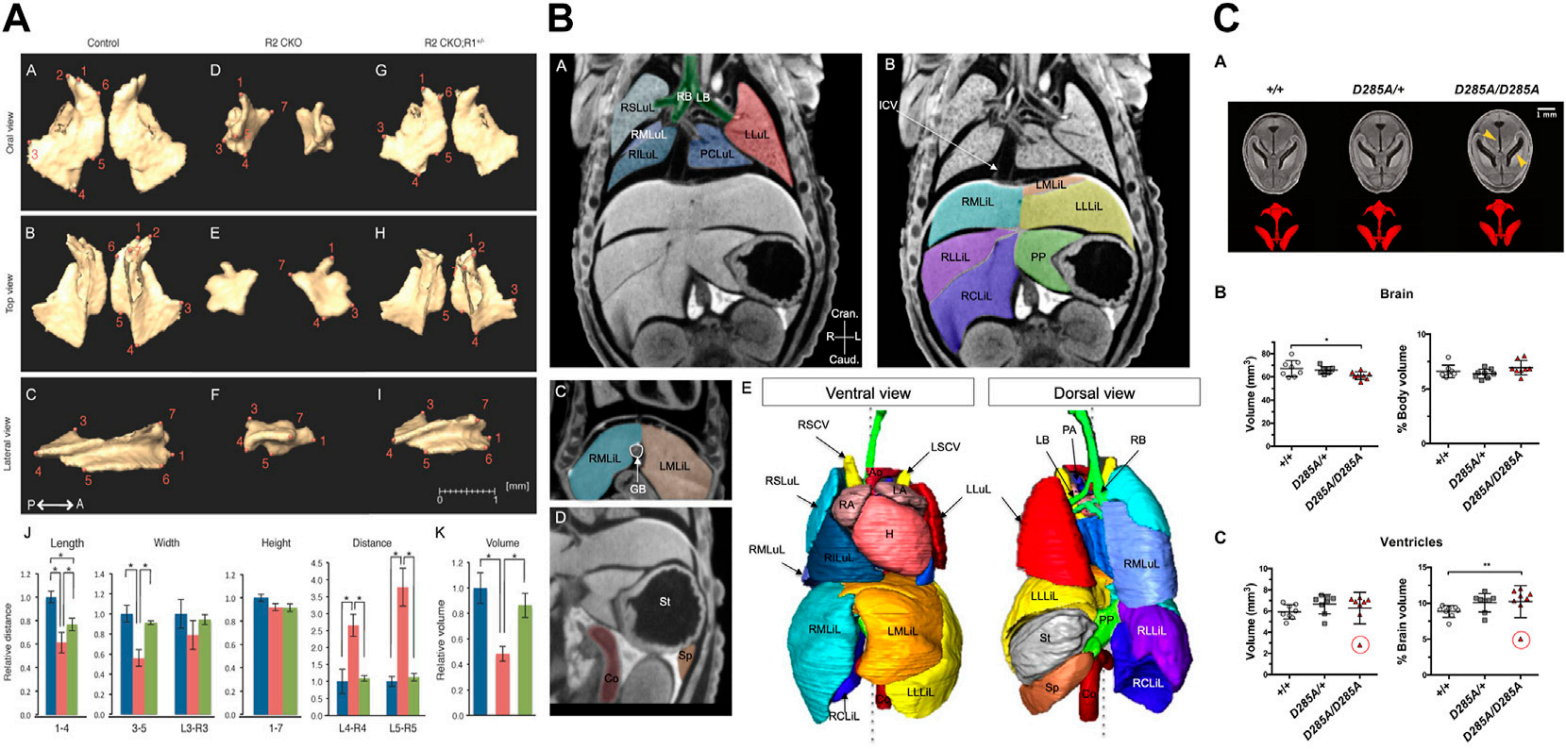
MicroCT for quantitative analysis of mutant mouse embryos. **(A)** Linear distance measurements and volume measurements quantify malformations in skull bones after loss of transforming growth factor beta (TGFβ) [Figure 5 from Ho et al., 2015, Developmental Biology 400 (2)]. **(B)** Image segmentation used for volumetry analysis of abdominal organs [Figure 3 from Desgrange et al., 2019, Disease Models & Mechanisms 12 (7)]. **(C)** Volumetry analysis of perinatally lethal homozygous *Oga* ^*D285A/D285A*^ mice showing defects in the brain and its ventricles (Figure 6 from Muha et al., 2021, Journal of Biological Chemistry 296). All images reprinted under the terms of the Creative Commons Attribution License.

In a very early study, Guldberg et al. (2004) measured the total volume of the mineralized skeleton at P2 and evaluated the dependence of volume measurements on voxel size and segmentation threshold. Later, a number of morphometric studies focused on developmental abnormalities of the skull. Jones et al. (2010) showed that loss of *Prkar1a* results in defects in intramembranous ossification including changes in bone volume and an absence of central palate bones, causing perinatal lethality. Rafiq et al. (2012) evaluated changes in the developing mandible between E15.5 and P21 by Thin-plate spline analysis and Procrustes analysis, and analysed deformations of the mandible in *Ror2* mutants at E18.5. Hill et al. (2014) showed that a conditional deletion of *Jagged1* leads to maxillary hypoplasia at P14, and used morphometry and densitometry measurements to compare bone structure and mineral density in the maxilla and palatine. Ho et al. (2015) investigated skeletal malformations in skull bones derived from cranial neural crest as a consequence of loss of transforming growth factor beta (TGFβ) by comparing length, width, height, volume, and surface area of the premaxilla, maxilla, palatine, frontal, and mandible (Figure 6A). Kawasaki et al. (2017) used length measurements to show that deficiency of *Bbs3* in Bardet-Biedl Syndrome (BBS) leads to a shorter palate in *Bbs3* ^*-/-*^ mice at E18.5. Marghoub et al. (2018) demonstrated that computational models accurately predict calvarial growth in both wild-type and mutant *Fgfr2* ^*C342Y/+*^ mice and in future may serve to manage clinically different forms of craniosynostosis. Kwon et al. (2018) analysed *Evc2* global mutants and found a smaller overall skull, and shorter nasal bone, frontal bone, and cranial base. Compared to skull development, fewer studies focused on the development of the post-cranial skeleton. Zhang et al. (2009) compared dose-dependent effects of *Runx2* on bone development by measuring different structural parameters of the tibia of newborns, including length and bone volume: total volume ratio in the metaphysis, and cortical thickness in the diaphysis. Moncayo-Donoso et al. (2019) analysed growth of the femur between E15.5 and P52 using measurements of femur length and thickness. Taken together, the microCT studies on skeletal development in prenatal and early post-natal stages showed that the protocols and instrumentation routinely used for quantitative analysis of the skeleton in adult mice can equally well be employed to quantify skeletal development.

MicroCT has also been used to quantitatively investigate the development of non-mineralized tissue. Merchant et al. (2016) measured the thickness of the myocardium of the left and right ventricle in hearts of E12.5 and E18.5 wild-type and *p27* ^*–/–*^ mutant mice. Desgrange et al. (2019) used microCT together with *in vivo* micro-ultrasound imaging and HREM in a standardized imaging pipeline for phenotyping laterality defects and associated heart malformations. The pipeline involved microCT for imaging at E18.5 and included segmentation of thoracic and abdominal organs and the great vessels (Figure 6B). The authors used segmentation-based volumetry analysis to evaluate the volume of the liver and spleen in *Rpgrip1l* ^*−/−*^ mutants, accompanied by qualitative evaluation of the vascular system, the position of abdominal organs, and the number of lobes of the liver and lung. Volumetry was also used by Muha et al. (2021) for the analysis of perinatally lethal homozygous *Oga* ^*D285A/D285A*^ mice at E 18.5, showing defects in the kidney, brain, liver, and stomach (Figure 6C).

### 4.3 Automated identification of phenotypes

**FIGURE 7:**
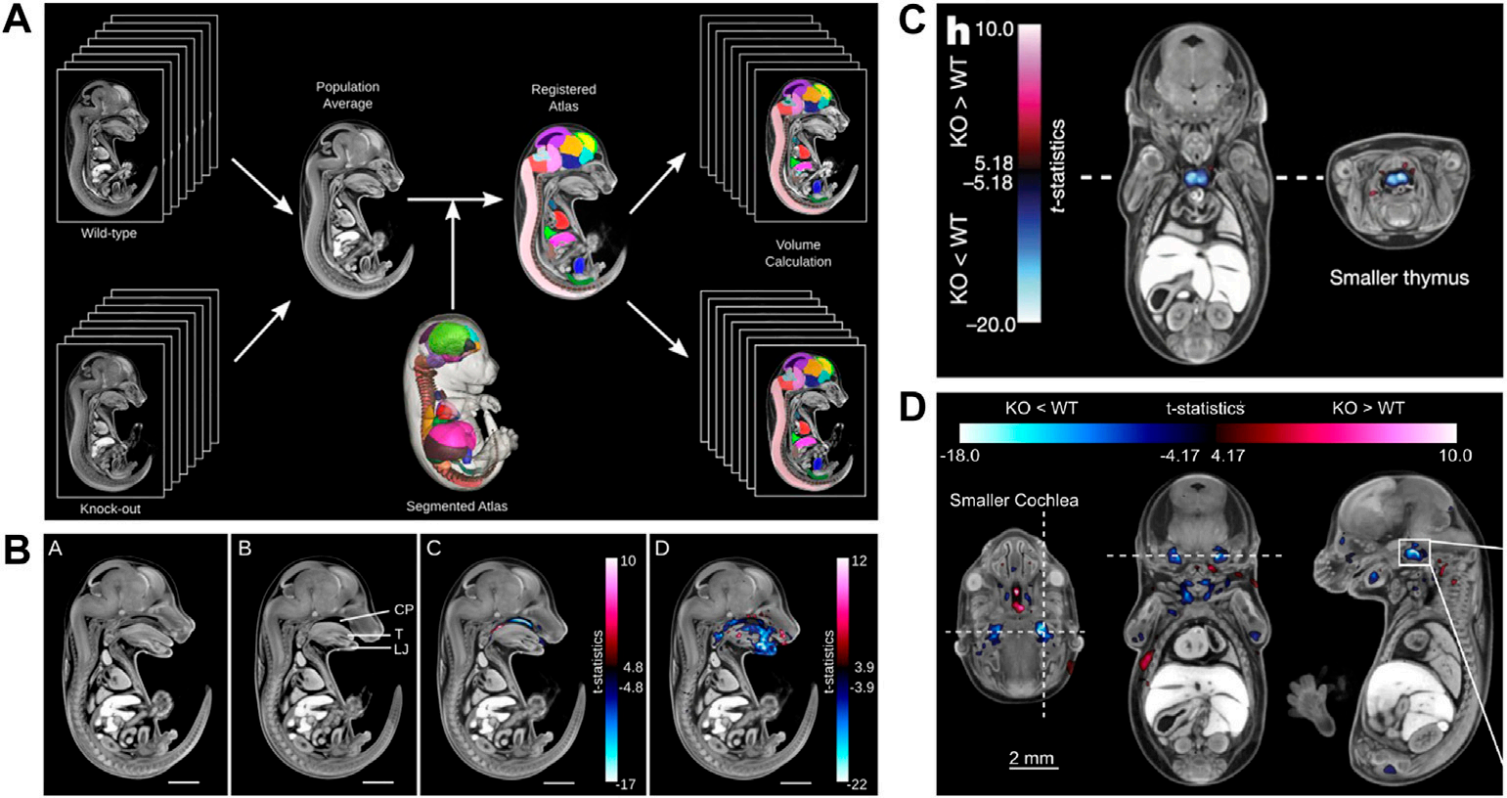
MicroCT for automated detection of novel phenotypes. **(A)** Automated image analysis pipeline using a microCT data-derived segmented mouse embryo atlas to detect developmental malformations [Figure 2 from Wong et al., 2014, Development 141 (12)]. **(B)** Automated detection of gross phenotypes by intensity- and deformation-based analysis in *Satb2* knockout mice (Figure 6 from Wong et al., 2014, Development 141 (12)). **(C)** Automated detection of subtle phenotypes, demonstrated on small thymus volume in E15.5 *Cbx4* knockout mice (parts of Figure3H from Dickinson et al., 2016, Nature 537). **(D)** Automated detection of subtle phenotypes, demonstrated on small cochlear volume in E15.5 *Eya4* mutant embryos (Extended Data Figure 6D from Dickinson et al., 2016, Nature 537). A and B reprinted by permission from the Company of Biologists. C and D reprinted by permission from Springer Nature.

Undoubtedly, the most significant contribution of microCT to phenotyping mouse development came from the IMPC screening project. This joint effort of numerous international research institutions achieved several major accomplishments and provided the first fully automated analysis pipeline to detect novel developmental phenotypes in mutant mouse lines. Prerequisites for the assay were the development of a reference atlas for E15.5 (Wong et al., 2012), a standardized sample preparation protocol (Wong et al., 2013b), and a fully automated image analysis pipeline for high-throughput screening (Wong et al., 2014). The workflow includes a combination of intensity-based, deformation-based, and annotated atlas-based image analysis, and enables to detect missing structures and volume differences in specific organs or in an entire embryo (Figures 7A,B). T-statistic maps combined with a false discovery rate (FDR) method for flagging phenotypes ensure that the probability of false positives is below 5%. In their proof-of-principle paper, Wong et al. demonstrated that the analysis pipeline captured both gross and subtle phenotypes. They used the hypomorphic *Tcf21* mutant mouse to show that in homozygous *Tcf21*-hypo embryos the analysis pipeline identified hypoplastic lungs, a narrower esophagus, and a smaller bladder. As a second example they showed that in *Satb2* knockouts their pipeline detected a missing palate, a shorter tongue and lower jaw, and several subtle defects such as e.g., missing primordia of the incisor teeth (Wong et al., 2014). The paper demonstrated the power of automatized approaches. By visual inspection of datasets, gross structural alterations such as a short lower jaw are likely detected, while subtle phenotypes such as a missing incisor primordium easily remain undetected.

The screening of 1751 knockout lines led to the discovery of numerous previously unknown developmental phenotypes (Dickinson et al., 2016). Of 283 lethal lines, many showed phenotypes before the embryos died. At E15.5, growth delay is the most common phenotype (>40%) followed by cardiovascular (>40%) and craniofacial (>20%) malformations (Dickinson et al., 2016). The large scale screening project also made use of the automated analysis pipeline of Wong et al. (Wong et al., 2014) to detect subtle phenotypes, such as a hypoplastic thymus in *CBx4* knockout mice (Figure 7C) or a smaller cochlea in *Eya4* mutants (Figure 7D) (Dickinson et al., 2016). In January 2022, the IMPC image database featured microCT scans of 257 mutants at E15.5 and 374 mutants at E18.5 (together with OPT scans from 65 mutants at E9.5, and 71 pre E.9.5 datasets). All MicroCT datasets are publicly available (https://www.mousephenotype.org/data/embryo) and can be viewed with an interactive embryo viewer or downloaded in medium and high resolution. For 164 of the E15.5 datasets a full volumetric analysis is available. Furthermore, the embryo viewer features annotations and false colour visualisations that allow scientists to interactively navigate through the datasets and explore differences to normal embryo structure. This makes the database a rich resource, which in future can be explored by new analysis tools such as modern deep learning segmentation routines. Very recently, the novel automated image analysis pipeline for mouse embryo phenotyping (LAMA) made use of the large amount of IMPC wild-type embryo control data to address issues of low mutant sample number as well as incomplete penetrance and variable expressivity (Horner et al., 2021). Employing a novel anatomical E14.5 embryo atlas with LAMA, it was possible to expose both known and novel dysmorphologies from two IMPC knockout lines.

## 5 MicroCT within the framework of contemporary microscopic 3D imaging modalities

The last two decades brought major technical advances in microscopic imaging. Established techniques were refined, new imaging modalities emerged. Imaging techniques suitable for monitoring mouse development fall into two categories. On the one hand, *in vivo* applications resolve cellular dynamics, track cell division and movement and map cell fate from pre-implantation to birth. *In vivo* imaging of mouse embryos is mainly performed using light-optical techniques such as confocal microscopy (CLSM), 2 photon microscopy (2PM), light sheet microscopy (LSM)/single plane illumination microscopy (SPIM), and OPT (Boot et al., 2008; Colas and Sharpe, 2009; Nowotschin et al., 2010; McDole et al., 2011; Udan et al., 2014; de Medeiros et al., 2016; McDole et al., 2018; Huang et al., 2020) but may involve ultrasound biomicroscopy, photoacoustic imaging, and optical coherence tomography (Norris et al., 2013). On the other hand, mouse embryos are assessed by a palette of *ex vivo* 3D phenotyping techniques, traditionally involving OPT, HREM, microMRI, and microCT (reviewed by Norris et al., 2013). The portfolio recently has been expanded by LSM/SPIM (Belle et al., 2014; Bunce et al., 2021). This section summarizes the strengths and limitations of microCT for mouse embryo phenotyping with regard to accessible specimen size and image resolution, signal specificity and molecular sensitivity, specimen preparation and shrinkage artefacts, and system availability and affordability. Because contemporary lab microCT systems are not suitable for studying cellular dynamics in embryos *in vivo*, the discussion will focus on a comparison of microCT to microMRI, HREM, OPT, and LSM/SPIM (summarized in Table 2).

**TABLE 2:** Comparison of contemporary *ex vivo* 3D microscopy modalities for mouse embryo phenotyping.

	MicroMRI	microCT	HREM	OPT	LSM
image contrast	excellent	excellent	excellent	excellent	excellent
voxel size (µm)	20-50	1-15	1-3	1-15	1-10
molecular sensitivity	medium (µM)	low (mM)	high (nM)	high (nM)	high (nM)
signal specificity	low-good	low-good	low-excellent	excellent	excellent
time window	> E10	> E5	any	<15	any
commercially available	yes	yes	yes	no	yes
sectioning	virtual	virtual	physical	virtual	optical

### 5.1 Accessible embryo size and image resolution

3D phenotyping of mouse embryos happens at the mesoscopic scale, i.e., at a spatial resolution between 2 and 50 µm (Wong et al., 2013a). MicroCT has been successfully used to image embryo sizes from E5.5 (Ermakova et al., 2018) to birth and beyond (Hsu et al., 2016) with voxel sizes ranging from 1 to 15 µm, depending on sample size. The largest amount of microCT data on mouse development has been generated for E15.5 and E18.5 (Dickinson et al., 2016). More recently automated analysis has been extended to E14.5 (Horner et al., 2021). In the IMPC screens, E15.5 embryos were imaged with a voxel size of ∼13 µm and datasets were downsampled to ∼28 µm before automated analysis (Wong et al., 2014), which allows to detect both gross and subtle phenotypes (Wong et al., 2014; Dickinson et al., 2016). MicroMRI has a more than two decades record in mouse developmental imaging (Dhenain et al., 2001). Due to physical limits for resolution, MRI is particularly efficient for the *ex vivo* phenotyping of the larger, late developmental stages (E15.5—birth). MicroMRI has been used for phenotyping of cardiovascular, pulmonary, palatal, and visceral anomalies in embryos at E15.5 at near isotropic voxel resolutions of ∼25 µm (Schneider et al., 2004; Szumska et al., 2008; Szumska et al., 2017) but voxel sizes down to 20 µm are feasible (Petiet et al., 2008). Developmental stages between E10.5 and E17.5 have been imaged by microMRI also *in utero*, albeit at lower isotropic voxel resolution (100 µm; Parasoglou et al., 2013; Zhang et al., 2018). In terms of voxel resolution, MRI data in phenotyping studies at E15.5 (∼25 µm) roughly equal downsampled IMPC datasets for E15.5 (28 µm). For both microMRI and microCT, phenotyping typically relates to confirming the presence of structure changes in tissues and organs. Yet, so far no systematic analysis has been conducted to evaluate the performance of microMRI versus microCT for the detection of phenotypes in different organs using conventional histology or HREM as ground truth. Such an analysis would help better understand the potential of the two modalities with respect to the detection of specific phenotypes at specific developmental stages. With HREM, a light-microscopic block-face technique providing high-contrast, high-resolution structural information (Weninger et al., 2018; Geyer and Weninger, 2019), voxel size is typically between 1 and 3 µm. Hence HREM clearly outperforms microMRI and microCT in resolution, and all developmental stages can be analysed. In the IMPC screenings, HREM has been used complementary to microCT to assess structural alterations, e.g., in the cardiovascular and nervous system, at histological resolution (Dickinson et al., 2016). Because of their higher resolving power, also fluorescence whole-mount imaging techniques such as OPT and LSM/SPIM are used for imaging early mouse embryos.

### 5.2 Image contrast, signal specificity and molecular sensitivity

MicroCT yields high image contrast between calcified and soft tissue (Paulus et al., 2001) and allows for accurate morphometric and densitometric measurements (Bouxsein et al., 2010). Consequently, for phenotyping the mineralized skeleton, microCT outperforms other microscopic imaging modalities. However, microCT yields low contrast in soft tissue. Therefore, compounds such as OsO_4_, I_2_KI, PTA, and PMA have been used for more than a decade to enhance embryo soft tissue contrast (Johnson et al., 2006; Metscher, 2009b; Descamps et al., 2014; Dickinson et al., 2016). Common to histological overview stains, these agents lack tissue specificity, enabling the inspection of general embryo anatomy only. While subtle differences exist in the staining patterns between OsO_4_, I_2_KI, PTA/PMA (Figure 3C), each compound is equally well suited to visualize embryo structure within the limits of resolution of microCT. Iodine-based contrast stains such as I_2_KI constitute the gold standard because they show lower toxicity (compared to, e.g., OsO_4_), are cheaper (compared to, e.g., OsO_4_), and penetrate more rapidly into tissue (compared to, e.g., PTA/PMA). Novel protocols specifically label cell nuclei (Müller et al., 2018; Metscher, 2020) and cytoplasm (Busse et al., 2018), providing an X-Ray dense analogue to the classic hematoxylin and eosin stain. While their applicability in embryo phenotyping pipelines has to be confirmed, specific stains for ubiquitous organelles (nuclei, cytoplasm) essentially will provide nonspecific stains at the tissue level, similar to the established compounds mentioned above. In comparison, microMRI, HREM, and OPT are also suitable to non-specifically reveal embryo anatomy. An advantage of microMRI is that it yields contrast in soft tissue already without prior staining, and this can be enhanced by the addition of contrast agents to the mounting medium (Schneider et al., 2004; Petiet et al., 2008). In HREM, high image contrast is achieved by adding eosin to the embedding medium (JB4-resin), yielding negative tissue contrast due to high background fluorescence of the resin (Weninger et al., 2018). In OPT, UV-triggered autofluorescence of the cleared embryo is exploited to visualize general embryo structure (Wong et al., 2013a; Dickinson et al., 2016) which also makes non-specific imaging possible without prior contrast treatment. Each of these techniques can be used for, e.g., visual inspection and detection of anomalies, automated detection of missing structures, or automated comparison of organ volumes or local volume differences.

All modalities are able to provide tissue- and molecule-specific contrast with the use of suitable contrast stains. Clearly, OPT and LSM/SPIM are currently the most powerful tools for molecule-specific embryo imaging, due to the wealth of available reporters including fluorochrome-labelled markers and endogenous fluorescence proteins. With tissue clearing, multi-channel fluorescence imaging is possible both at the microscopic and mesoscopic scale. LSM/SPIM has been used *ex vivo,* e.g., for the phenotyping of axonal tracts (Belle et al., 2014), for imaging of PAX8 expression in the developing urogenital system (Bunce et al., 2021), and for high-resolution imaging of embryonic vasculature (Renier et al., 2014). OPT is especially versatile as it can be used for imaging both reflected light (e.g., fluorescing reporters, for visualizing gene expression patterns) and transmitted light (brightfield imaging, e.g., lacZ expression; Sharpe et al., 2002; Sharpe, 2003). OPT data contribute to the EMAGE mouse embryo spatial gene expression database (Richardson et al., 2010; Richardson et al., 2014). While this refinement in compound-specific labelling is not yet available to microCT investigation, recent advances in sample preparation allow for cell- and tissue-specific labelling that is detectable and promise the possibility of molecular analysis of mouse embryos by microCT, including the systematic examination of tissue-specific effects of mutations. For example, while the nonspecific staining with iodine as used in the IMPC screenings gives excellent contrast to most embryonic tissues, it fails to discriminate reliably some types of cartilage from surrounding connective tissue. Also, iodine staining tends to decalcify bone (Heimel et al., 2019) complicating the quantitation of bone mineral particularly in intensity-based analyses of minute embryonic mineralisations. Recently, Gabner et al. (2020) presented a selective staining protocol for embryonic cartilage using ruthenium red that leaves bone mineral unaffected and allows, for the first time, imaging of the cartilage skeleton of an entire embryo with sufficient contrast to conduct intensity-based visualisation and analysis. This protocol could be used to build reference atlases of the embryonic skeleton at specific developmental stages, which may then be used to automatically detect structural deviations similar to automated phenotyping conducted in the IMPC analysis pipeline (Wong et al., 2014). MicroCT image acquisition could also be adapted to monitor gene expression systematically. For example in most IMPC alleles, a lacZ cassette is present and lacZ expression has been routinely evaluated in the IMPC screenings in heterozygous embryos at E12.5 using classical reporter visualization with X-gal staining, clearing in glycerol, and 2D light microscopy (Dickinson et al., 2016). Very recently, Ermakova et al. (2021) demonstrated that the blue and insoluble chromogenic product of the X-gal reaction, based on its content of bromine atoms, is X-ray dense, providing an effective and microCT compatible label of lacZ activity *in situ*. It is tempting to envision that high-throughput phenotyping of mutant animals also involves the systematic microCT analysis of lacZ reporter expression (as done before by brightfield OPT) and its volumetric localization to specific anatomical structures. Evidently, such a 3D quantitative assay could take advantage of all automated processing tools for distortion correction, 3D reconstruction, registration, and relative quantitative analysis (e.g., Wang et al., 2021). Despite expected advances in exogenous contrast agents and new capabilities for biomarker detection, the inherently low molecular sensitivity of microCT (mM range) compared to OPT and LSM/SPIM (nM range; Walter et al., 2020) will limit its potential for the analysis of molecular signals. Routine microCT screens for lacZ expression patterns in transgenic mouse lines would therefore require a thorough evaluation of the sensitivity of the assay beforehand.

### 5.3 Specimen preparation and shrinkage artefacts

A major limitation of contrast-enhanced microCT is soft tissue shrinkage during specimen preparation, a problem common to microscopic *ex vivo* techniques (Rodgers et al., 2021). Several factors contribute to artifactual tissue volume changes. Their effects are difficult to discern, frequently unknown, and yet relevant for data interpretation. In addition, conditions of microCT specimen preparation widely differ depending on application and experimenter. Together, this hampers the comparison of morphometric data deriving from different studies. Micro-CT imaging requires fixation and staining with heavy chemical elements to render soft tissue visible (see also 3.1, 3.2). Tissue volume changes caused by fixation depend on the chemical nature of the fixative (formaldehyde, glutaraldehyde, ethanol), the volume and composition of the fixation buffer (pH, osmolarity), and fixation time, among other parameters (Vickerton et al., 2013). For example, formaldehyde causes up to 12% tissue shrinkage which can be reduced to 5% using lower concentration fixative (Hedrick et al., 2018). Further shrinkage occurs during contrast staining. The three most popular microCT stains (I_2_KI, I_2_ in absolute ethanol, PTA) deliver volume shrinkage ranging from, e.g., 10–56% for adult muscle tissue and 27–66% for adult brain tissue (Buytaert et al., 2014). Thus, shrinkage during staining differently affects different tissues. Shrinkage in iodine-based contrast agents is higher than in PTA (Buytaert et al., 2014; Lesciotto et al., 2020). Stabilization of embryos in polymerizing hydrogels prior to iodine staining reduces shrinkage and sample deformation (Wong et al., 2013a; Hsu et al., 2016; Hsu et al., 2019). Interestingly, quantified statements on effects on shrinkage are missing even though hydrogel infiltration is part of the specimen preparation routine in the IMPC phenotyping pipeline. While it is possible, that uncontrolled shrinkage of fetal tissues may obscure malformations or induce artefacts, which erroneously could be interpreted as malformations (Dawood et al., 2021), the microCT data generated by the IMPC still may be considered robust for phenotyping because hydrogel embedding minimizes intersample variation (Wong et al., 2013b), suggesting that shrinkage is comparable between specimens. Together, the available data point to the possibility that the combination of formaldehyde-fixation, hydrogel-embedding (Wong et al., 2013b) and neutral-buffered iodine infiltration (Dawood et al., 2021) could largely eliminate tissue shrinkage in mouse embryos for microCT examination, but this needs to be evaluated in future studies. For morphometric data of iodine-stained mouse embryos not infiltrated with hydrogel prior to staining (see e.g. Desgrange et al., 2019; Muha et al., 2021), it must be assumed that volume measurements clearly underestimate *in vivo* organ volumes and therefore adjustment of the results may be necessary for comparison with IMPC data.

Other imaging modalities similarly suffer from artefacts by specimen processing. In HREM, shrinkage is caused by dehydration and JB4 resin embedding but quantified data are missing (Reissig et al., 2021). In LSM/SPIM, clearing may cause massive shrinkage, the extent depending on the type of agent (BABB, Spalteholz fluid, 3DISCO; Miller et al., 2005; Buytaert et al., 2014; Azaripour et al., 2016; Vigouroux et al., 2017; Vulders et al., 2021). MicroMRI yields high-contrast images from unstained soft tissues (Zamyadi et al., 2010) that are not devoid of artefact but the advantage over microCT is that volume shrinkage derives from fixation only and therefore is smaller and easier to evaluate. In contrast-enhanced microMRI analyses of mouse developmental stages (Schneider et al., 2004; Zamyadi et al., 2010), the effects of gadolinium (added to the mounting medium) on tissue volume changes have not been evaluated so far. In conclusion, there is a clear need for i) the mandatory and precise reporting of experimental conditions during sample preparation including information on parameters such as concentration, pH, osmolarity, temperature, volume of contrast staining solutions, duration of treatments, and conditions of incubations (media changes), ii) the implementation of relevant detail into the protocols of standardized phenotyping pipelines, and iii) the rigorous quantification of the effects of experimental parameters on volume changes.

### 5.4 Analysis and reporting on sensitivity and specificity

Sensitivity (true positive rate) and specificity (true negative rate) describe the accuracy of any test reporting the presence or absence of a condition. So far, both measures have not been systematically explored in studies using microCT for mouse developmental phenotyping, which is different to MRI (Table 3 in Adams et al., 2013). Due to the high contrast and resolution in microCT images of stained embryos, it has been generally assumed that the sensitivity for detecting phenotypes is high. Kim et al. (2013) explored sensitivity and specificity of their microCT assay for detecting different congenital heart diseases in iodine-stained neonates and foetuses using histopathology as ground truth. The authors reported a sensitivity of 85.7% and a specificity of 91.6% for ventricular septal defects, corresponding to an accuracy of 89.8%. Since sensitivity and specificity were higher for detecting other structural anomalies (>97% accuracy for several investigated diseases including outflow tract and aortic arch anomalies), the authors concluded that microCT reliably detects a wide spectrum of CHD and can thus be used for routine assessments (Kim et al., 2013). However, sensitivity and specificity only can be determined for specific experimental situations. The measures are expected to vary depending on the protocol and equipment utilized. More data on the variance of sensitivity and sensitivity under standardized and comparable conditions of sample preparation and imaging are needed and will help get a clearer picture as to whether high accuracies of >95% can be generally expected for morphological features. Finally, as discussed in Section 5.1, it will be informative to compare the sensitivities and specificities of microCT, microMRI, HREM, and OPT for a specific phenotype at a specific developmental stage.

### 5.5 System availability and costs

Commercial laboratory microCT scanners are quite affordable and available from various vendors. Over the last 15 years the number of microCT systems at universities and research institutions constantly increased, making microCT the most widely and easily available modality for 3D phenotyping of mouse embryos. OPT currently is not commercially available. Setups are custom built from affordable components (Wong et al., 2013a; Ramirez et al., 2019), hence are rare and not routinely available to researchers. Still, OPT contributed to the IMPC screens (Dickinson et al., 2016) and the EMAGE database (Richardson et al., 2014). HREM was initially performed on custom setups and only recently became commercialized by a single vendor (Reissig et al., 2021; Walsh et al., 2021). To date, the availability of HREM to researchers is limited and HREM is less frequently used than microCT. Ultra-high-field microMRI systems for embryonic phenotyping require field strengths of 10 Tesla or higher (Schneider et al., 2004) and therefore are costlier than microCT in purchase and maintenance. The relatively higher running costs for microMRI scans can be reduced by mounting multiple specimens in a tube (Schneider et al., 2004), enabling to scan up to 40 embryos in a 12 h run (Norris et al., 2013).

## 6 Outlook

The first microCT image of a mouse embryo was published in 1998 (Sasov and van Dyck, 1998). Since then, among several modern 3D imaging modalities microCT has grown to become a key technique for gathering qualitative and quantitative data on mouse embryo structure, both non-invasively and at microscopic resolution (Dickinson et al., 2016). Beyond that, recent advances in sample preparation and image acquisition hold promise that there is potential for a more important role of microCT in embryonic phenotyping.

### 6.1 MicroCT as a bridge technique in correlative multimodal imaging

**FIGURE 8:**
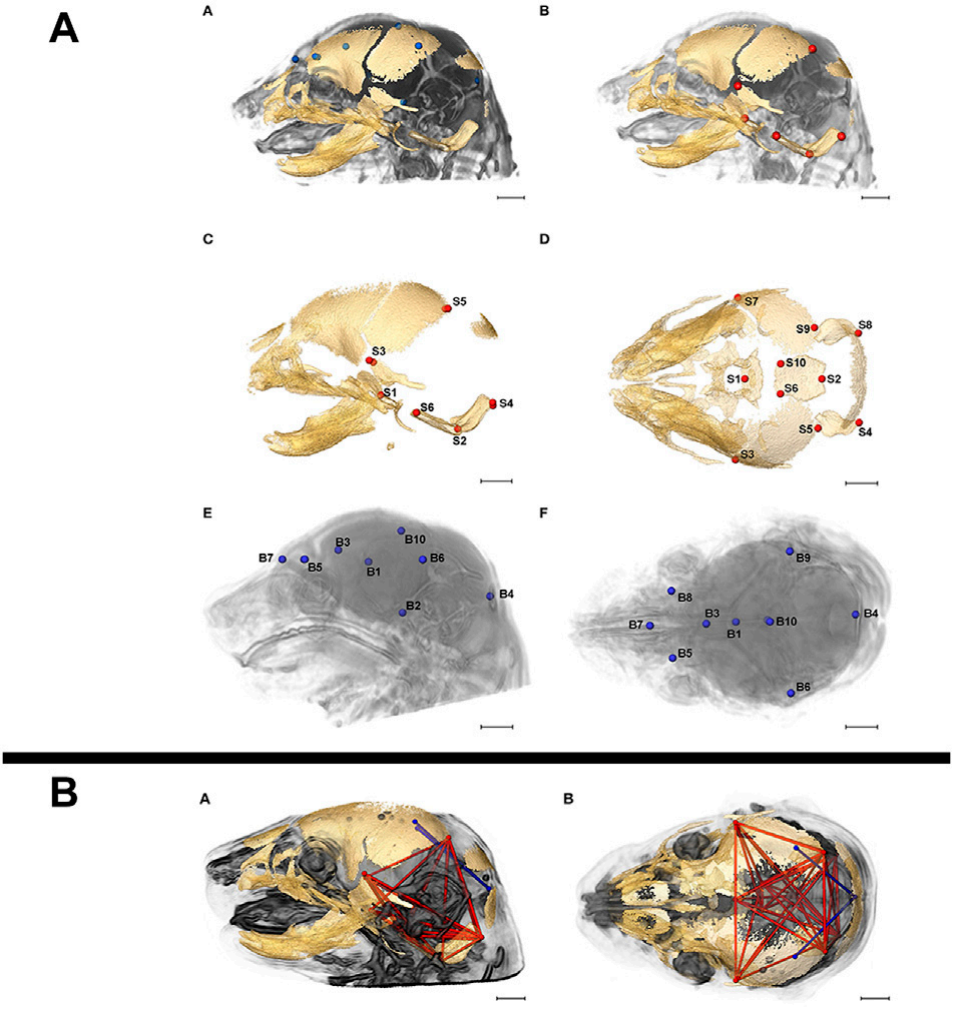
Correlative imaging of mouse embryos with microCT and MRI. **(A)** Skull and brain landmarks set on co-registered microCT and high-resolution MRI data sets. The inter-landmark distances are used in a PCA analysis to assess the integration of skull and brain development. MicroCT reveals bone structure, MRI retrieves brain morphology in formaldehyde-fixed but otherwise uncontrasted E17.5 mouse embryos (Figure 2 from Motch Perrine et al., 2017, Frontiers in Human Neuroscience 11). **(B)** Brain (blue) and skull (red) linear distances with statistically significant different association between *Fgfr2c* ^*C342Y/+*^ Crouzon syndrome mice and *Fgfr2* ^*+/S252W*^ Apert syndrome mice at E17.5 (Figure 6 from Motch Perrine et al., 2017, Frontiers in Human Neuroscience 11). All images reprinted under the terms of the Creative Commons Attribution License.

Due to its non-destructive nature, microCT can easily be implemented in correlative multimodal imaging (CMI) workflows where the same sample is analysed consecutively by different imaging modalities, either across scales to gain a more complete understanding of structure or across methods to collectively retrieve structural and molecular information (Walter et al., 2020). MicroCT and high-resolution magnetic resonance imaging (MRI) have been combined to resolve bone structure and brain morphology, respectively, in analyses of skull and brain development in prenatal mice (Figure 8) (Luo et al., 2017; Motch Perrine et al., 2017), and in several studies, structural phenotypes established by microCT have subsequently been confirmed by histological examination (Dickinson et al., 2016).

For CMI pipelines that bridge macroscopic with microscopic imaging, usually mesoscopic resolution assays need to be implemented. In this context, microCT has been used as an intermediate imaging technology to support the integration of datasets by creating a 3D template of the sample before sectioning. MicroCT reveals tissue structure in thick samples in 3D at micrometer resolution, tracks distortions and morphological changes of the ROI after embedding and fixation, and allows ROI identification for downstream analyses. MicroCT is specifically suited as an intermediate technology in correlative light and electron microscopy (CLEM) pipelines since it can visualize the sample embedded in resin blocks due to the heavy-metal stains used for EM sample preparation (Handschuh et al., 2013; Karreman et al., 2016; Karreman et al., 2017). It also qualifies for correlative microscopy approaches where the visualization of exogenous (e.g., metal beads) or endogenous landmarks (e.g., vasculature, after perfusion of contrast agent) is required. The bridge role of microCT so far has not yet been exploited systematically for embryonic phenotyping, e.g., the improved and faster targeting of identified dysmorphic structures for subsequent detailed microscopic and ultrastructural analysis.

### 6.2 MicroDECT for routine phenotyping of mutant mouse embryos

**FIGURE 9:**
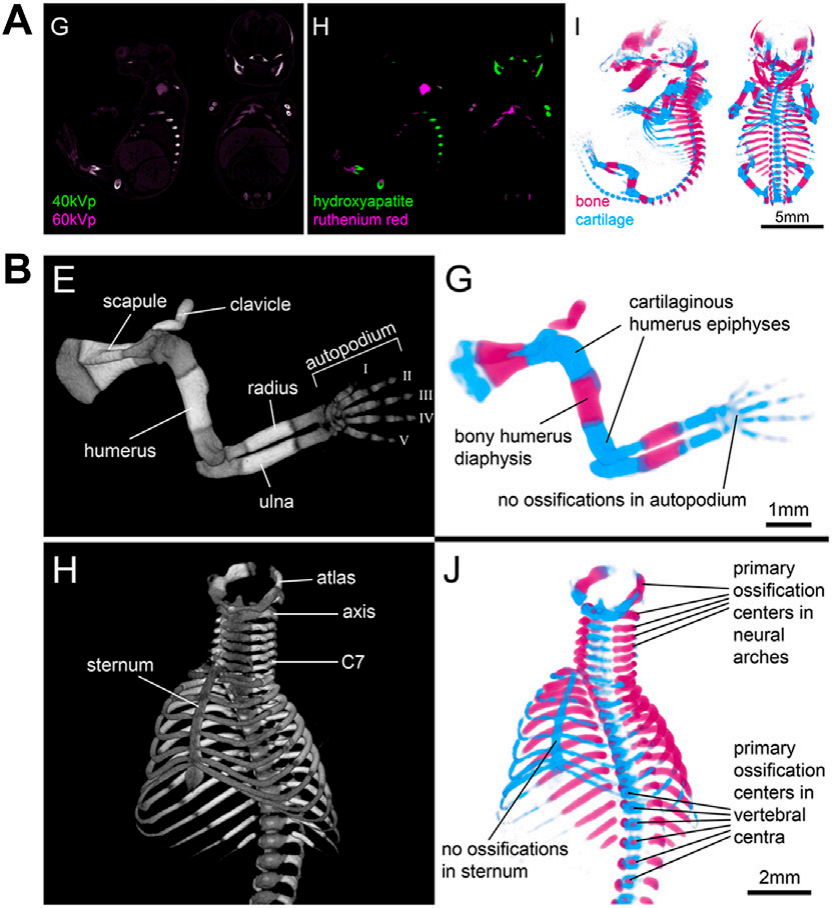
Microscopic dual energy CT (microDECT) for imaging skeletal development. **(A)** MicroDECT for imaging cartilage and bone in ruthenium red stained E16.5 mouse embryos. The embryo is scanned using two different X-ray spectra (40 kVp, 60 kVp), which allows separating of hydroxyapatite and ruthenium attenuation using material decomposition. Material fractions can be visualized using volume rendering and false colours mimicking the colour of Alizarin red/Alcian blue stained specimens (Figures 3G–I from Gabner et al., 2020, Development 147 (11)). **(B)** Examples of conventional greyscale microCT visualisation (left) and microDECT visualisation (right) of the developing forelimb and ribcage in a wild type E16.5 mouse embryo [Figures 4E,G,H,J from Gabner et al., 2020, Development 147 (11)]. All images reprinted by permission from the Company of Biologists.

Finally, the potential of spectral imaging approaches such as microscopic dual energy CT (microDECT) has not yet been fully explored for developmental imaging. MicroDECT yields multi-channel CT volumes of multiply contrasted specimens at micron resolution, provided the elemental k-edges of the x-ray dense compounds are sufficiently far apart to achieve a strong spectral contrast (Johnson, 2012). For example, established soft tissue stains such as I_2_KI and PTA can be separated in double-stained specimens (Handschuh et al., 2017). Since I_2_KI and PTA largely nonspecifically contrast tissue and deliver images resembling classical histological overview stainings, it is desirable to employ microDECT with combinations of nonspecifc and tissue-specific contrast agents. In a proof-of-principle study, Gabner et al. demonstrated the separation of ruthenium red stained cartilage and bone in E16.5 mouse embryos (Figure 9), making possible the systematic and quantitative examination of the entire developing skeleton for the first time (Gabner et al., 2020). Future studies will be needed to explore the potential of double-labelling protocols and microDECT to discriminate further tissue- and organ specific probes and test their suitability for embryonic phenotyping.
